# P02.96. Effects of deep breathing on pulse pressure harmonics

**DOI:** 10.1186/1472-6882-12-S1-P152

**Published:** 2012-06-12

**Authors:** W Li, A Ahn

**Affiliations:** 1 Massachusetts General Hospital, Charlestown, USA

## Purpose

Pulse pressure waveforms can be decomposed into individual harmonics using frequency-domain analyses. Based on the well-recognized phenomenon of pulse wave reflections, these harmonics are purported to play important physiological functions and form the basis for Traditional Chinese Medicine (TCM) pulse diagnosis. Although breathing is considered an essential part of TCM and Qi Gong, its effects on pulsatile hemodynamics are unknown. This study explored the effects of slow, deep breathing on blood pressure harmonics.

## Methods

We recruited 15 healthy subjects and measured blood pressure with piezo-electric finger pulse transducers during normal and slow deep breathing. A total of 29 epochs of alternating normal and deep breathing maneuvers were obtained. The pressure waveforms were analyzed with an enhanced Morlet wavelet transformation using DataDemon software. The frequencies and amplitudes of the blood pressure harmonics (from 1^st^ to 6 ^th^) were compared between normal and deep breathing.

## Results

During normal breathing, the mean modulus amplitudes for the 1^st^ to 6^th^ harmonics were 0.037, 0.028, 0.03, 0.015, 0.01, and 0.008. With deep breathing, the mean amplitudes were decreased across the six harmonics compared to normal breathing with a 12%, 17%, 25%, 15%, 16%, and 15% reduction in the 1^st^ to 6^th^ harmonics, respectively. A two-tailed paired t-test revealed statistically significant reductions in the moduli for the 2^nd^ and 3^rd^ harmonics (p=0.03 for both) but not for the others. Despite these trends, the relative amplitudes of the harmonic moduli were significantly changed when different breathing techniques were performed.

## Conclusion

Relaxed deep breathing is associated with significant reductions in the 2^nd^ and 3^rd^ pulse wave harmonics. The physiological importance of these changes is unclear but may help elucidate the physiological basis of pulse diagnosis.

